# Continuous‐Wave (CW) Photo‐CIDNP NMR Spectroscopy: A Tutorial

**DOI:** 10.1002/mrc.70031

**Published:** 2025-09-04

**Authors:** Lars T. Kuhn, Míriam Pérez‐Trujillo

**Affiliations:** ^1^ Institut für Physikalische Chemie Albert‐Ludwigs‐Universität Freiburg Freiburg i. Br. Germany; ^2^ Servei de Ressonància Magnètica Nuclear, Facultat de Ciències i Biosciències Universitat Autònoma de Barcelona Cerdanyola del Vallès Catalonia Spain

**Keywords:** hyperpolarisation, in situ spectroscopy, NMR, photochemistry, photo‐CIDNP

## Abstract

Photo‐chemically induced dynamic nuclear polarisation (photo‐CIDNP) is a nuclear spin‐selective magnetic resonance phenomenon that has traditionally been used to mechanistically study chemical reactions involving the (transient) formation of radical molecular species, extract EPR observables of short‐lived radicals, probe biomolecular structure and interactions and, less importantly, increase the sensitivity of a nuclear magnetic resonance (NMR) measurement. Recently, the introduction of significant methodological advances as well as the advent of benchtop NMR spectroscopy has rekindled interest in this technique, which—serendipitously discovered more than half a century ago—has, as of late, matured into a powerful, highly sensitive and extremely versatile NMR hyperpolarisation method. In this tutorial, aimed primarily at the nonexpert user, we provide practical information on how to plan, set up and perform one‐dimensional ^1^H and heteronuclear photo‐CIDNP NMR experiments using a high‐field NMR spectrometer and a continuous‐wave (CW) illuminant. In particular, strategies for selecting the appropriate experimental setup are described, including aspects such as light source requirements, introduction of appropriate light coupling methods and photosensitiser selection. In addition, examples of suitable one‐dimensional ^1^H and heteronuclear photo‐CIDNP pulse schemes are presented, photo‐CIDNP‐specific acquisition parameters—including the implementation of sequence commands required to trigger the light source—are explained, and ‘hands‐on’ practical advice on photo‐CIDNP sample preparation is provided. Finally, special attention as to how to acquire and analyse one‐dimensional photo‐CIDNP data in a meaningful way is given.

## 1 Introduction

**FIGURE 1:**
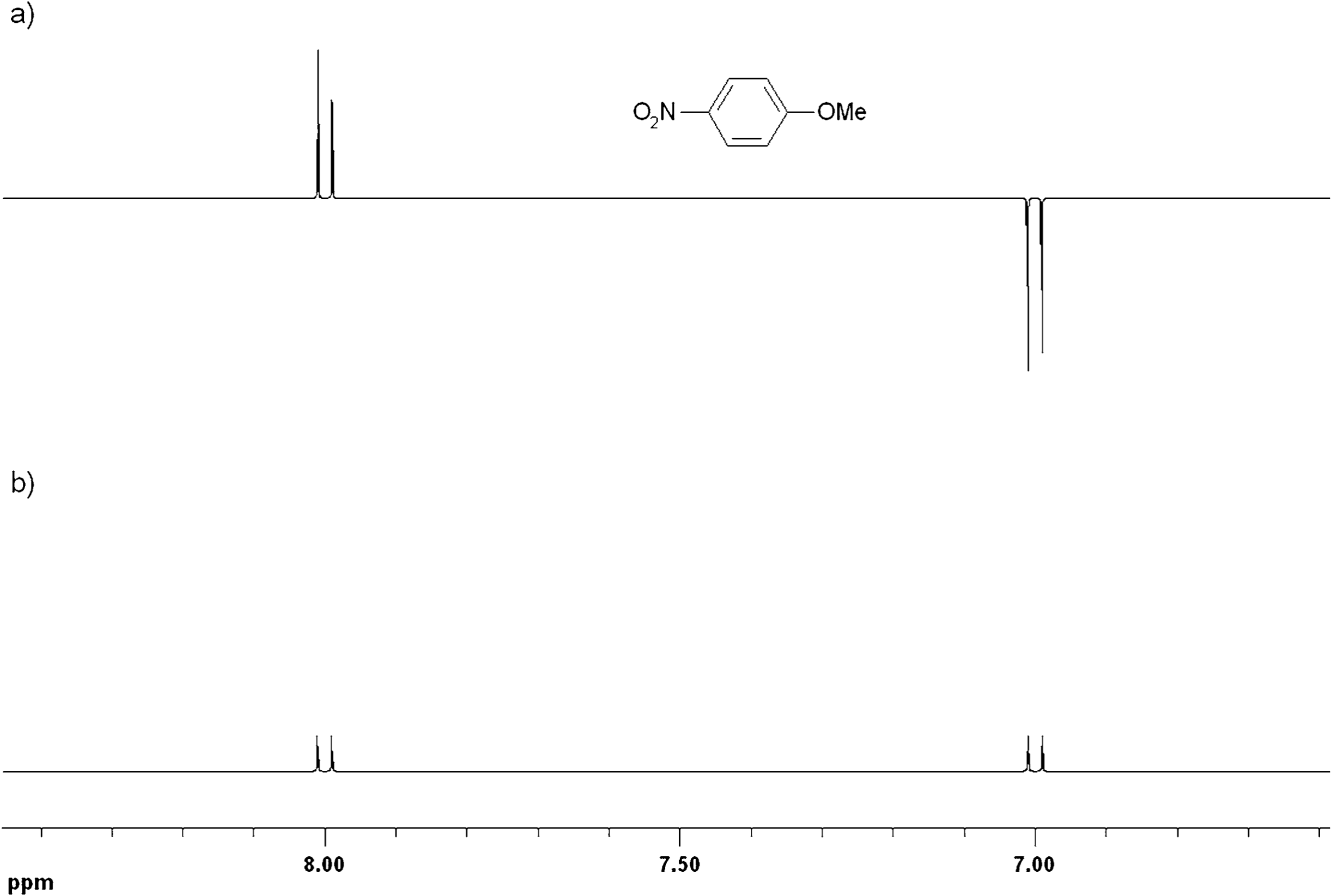
(a) ^1^H‐CIDNP NMR spectrum (aromatic region) acquired ‘in situ’ during the reversible addition of pentafluorobenzoyloxy radicals to 4‐nitroanisole featuring both absorptively enhanced and emissive polarisation signals representing the aromatic *ortho*‐ and *meta*‐protons, respectively; (b) ^1^H NMR spectrum of the same reaction mixture acquired ca. 5 min after the end of the reaction.

**FIGURE 2:**
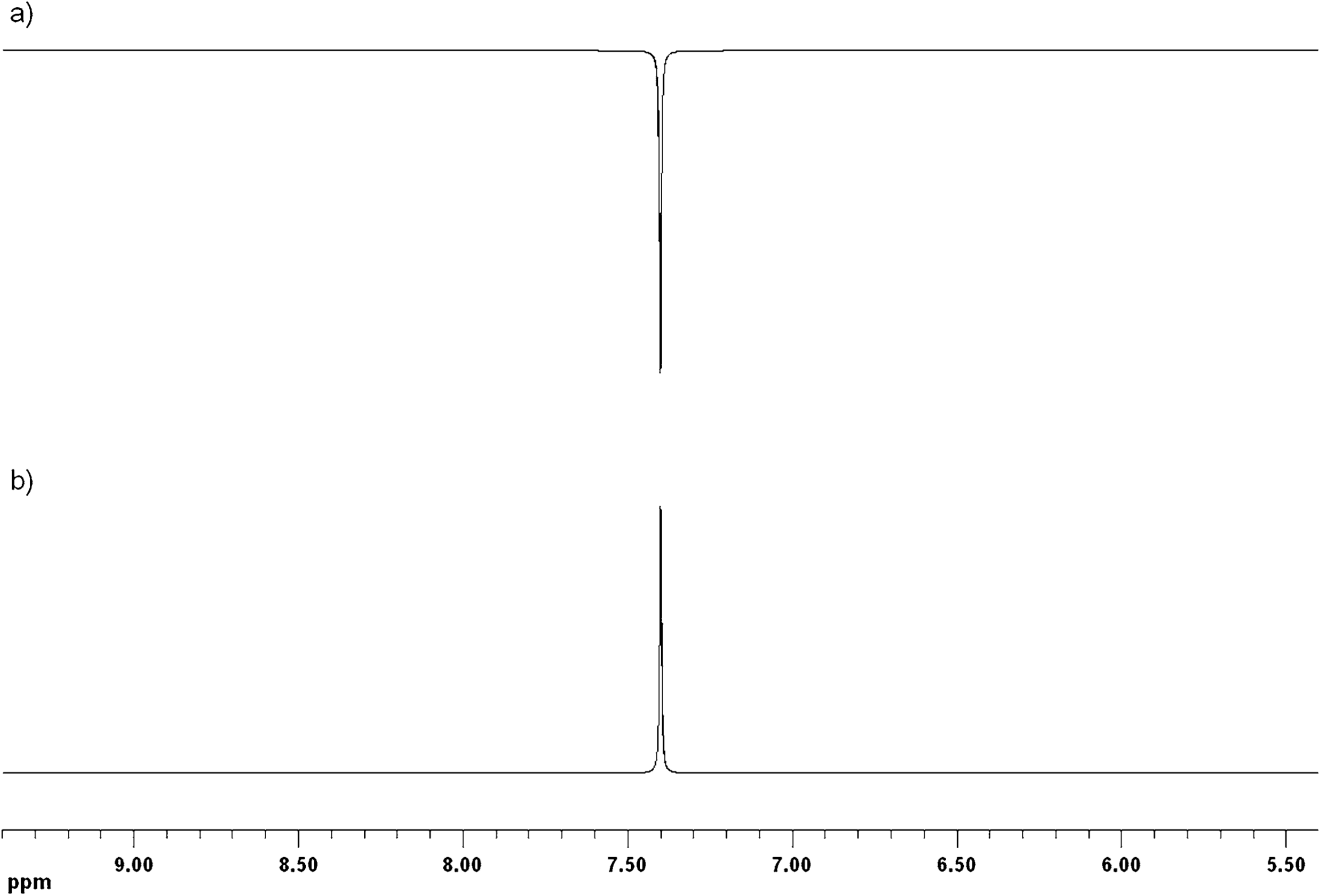
(a) ^1^H‐CIDNP NMR spectrum acquired ‘in situ’ during the reversible addition of pentafluorobenzoyloxy radicals to benzene. The emissive signal at ca. δ = 7.4 is the resonance line of benzene; (b) ^1^H NMR spectrum of the same reaction mixture acquired ca. 5 min after the end of the reaction.

The serendipitous observation of anomalously emissive nuclear magnetic resonance (NMR) signals during the thermally induced decomposition of dibenzoyl peroxide (DBPO) made by Bargon et al. some 60 years ago [1, 2] marked the discovery of a magnetic resonance phenomenon that, shortly afterwards, became known as chemically induced dynamic nuclear polarisation, or simply CIDNP. Remarkably, the effect, which was independently observed almost simultaneously by Ward and Lawler [3], manifests itself as emission and/or enhanced absorption in the NMR signals of the products of radical reactions in solution carried out in the presence of a magnetic field (Figures 1 and 2) and arises from the ability of magnetic nuclei to modulate the electronic spin state of a radical pair.

**FIGURE 3:**
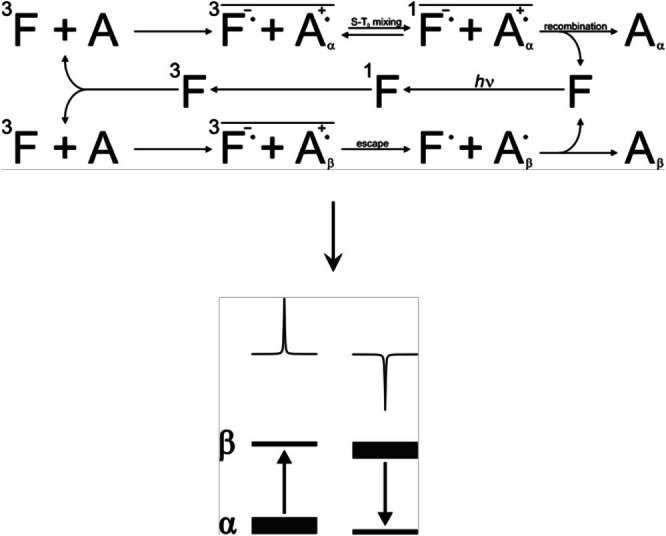
Top: representation of the cyclic photochemical reaction scheme responsible for the formation of photo‐CIDNP in small (bio‐)molecules (A), e.g., amino acids, via the radical pair mechanism (RPM), using a flavin‐based photosensitiser (F). An electron transfer mechanism is shown; a similar set of reactions takes place if the initial step is hydrogen abstraction. The overbar indicates spin‐correlation. Bottom: liquid‐state photo‐CIDNP leads to the observation of enhanced absorptive or emissive NMR signals.

Early theories attempting to interpret and explain CIDNP qualitatively and quantitatively [1, 2, 4, 5] were based on mechanisms responsible for the nuclear Overhauser effect (NOE) and dynamic nuclear polarisation (DNP). However, they all failed to explain both the magnitude of the detected enhancements and the observed signal patterns. Shortly thereafter, an alternative explanation was proposed by Closs [6] and independently by Kaptein and Oosterhoff [7], the so‐called CKO model, which explained all the experimental observations much more convincingly. Their theory emphasised the ability of magnetically active nuclei to alter the electronic spin state of a radical pair, thereby modulating its chemical reactivity. This theoretical approach subsequently became known as the radical pair mechanism (RPM) and led to the application of both thermally and photochemically induced CIDNP to mechanistic problems in organic chemistry, as the observed NMR enhancements could be utilised to identify and elucidate the pathways of reactions that proceed via one or more radical pair intermediates (see Section 2). Thus, the CIDNP effect was employed early on for the mechanistic study of chemical reactions involving elusive (organic) radicals, including the extraction of some of their EPR parameters [8, 9, 10, 11]. In 1978, Kaptein proposed a CIDNP technique in which polarisation is generated in cyclic photochemical reactions between an excited photosensitiser and certain aromatic amino acid side chains present on the surface of a protein (Figure 3) [12], thus monitoring their solvent accessibility or ‘exposure’. Since its first application, biomolecular ‘photo‐CIDNP’ has proven to be a powerful probe of protein structure and of the many factors that modify the solvent‐exposure of polarisable amino acid residues [13, 14]. Common applications included investigations of protein interactions with cofactors, inhibitors, nucleic acids, lipids and other proteins, comparisons between related proteins, and, importantly, studies of conformational changes and denaturation both in equilibrium and in real‐time as a protein folds or unfolds ‘in situ’, i.e., inside an NMR tube in the spectrometer [15, 16, 17].

Over the last 15 years or so, Cavagnero and co‐workers have introduced further methodological improvements to increase the applicability of the biomolecular photo‐CIDNP effect [18, 19, 20, 21]. Importantly, these have mainly focused on boosting its sensitivity, a feature that the method had always lacked as compared to other hyperpolarisation techniques, e.g., parahydrogen‐induced polarisation (PHIP) or DNP. In addition, the availability of inexpensive light sources [22, 23], the introduction of novel sample illumination methods [24] and the development of new applications of photo‐CIDNP both in biomolecular fragment screening of pharmacologically relevant leads [25] as well as in metabolomics [26] have rekindled interest in this technique, which—complemented by the widespread introduction of benchtop NMR spectrometers [27]—has developed into a powerful, highly sensitive and extremely versatile NMR hyperpolarisation method.

In this tutorial, we attempt to shed light on how to practically plan, set up, and conduct one‐dimensional ^1^H and heteronuclear photo‐CIDNP NMR experiments using a state‐of‐the‐art NMR spectrometer in combination with a continuous‐wave (CW) illuminant. In particular, strategies for identifying a suitable experimental setup are presented, including relevant aspects such as light source requirements, implementation of a suitable light guide and selection of appropriate fibre coupling methods. In addition, efficient one‐dimensional photo‐CIDNP pulse schemes are presented, photo‐CIDNP‐specific sequence parameters are explained, and practical advice is given on the preparation of standard photo‐CIDNP samples, including the use of a suitable photosensitiser. The tutorial concludes with a special focus on how to acquire, process and analyse photo‐CIDNP data in a meaningful way. Time‐resolved (TR) photo‐CIDNP NMR, a lower sensitivity variant of the method which—in contrast to continuous‐wave photo‐CIDNP—employs much shorter, i.e., nanosecond‐long, high‐power laser pulses and is mainly used to study the electronic structure, e.g., hyperfine interaction networks, of transient radicals, as well as solid‐state photo‐CIDNP methods are not discussed here. These experiments, extensively discussed and reviewed elsewhere in the literature [21, 28], afford specific, conceptually very different setup requirements as compared to continuous‐wave photo‐CIDNP NMR spectroscopy performed in liquids.

## 2 Theoretical Background—The Radical Pair Mechanism (RPM)

**FIGURE 4:**
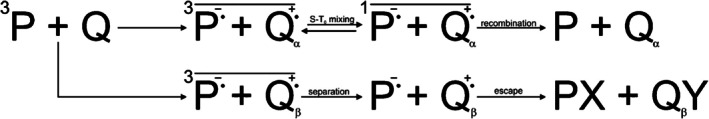
Schematic representation of the radical pair mechanism (RPM) and the spin sorting process. In this example, singlet–triplet mixing is faster when the proton on Q is in the α‐state. The overbar indicates the spin‐correlation between the two radical partners.

The CIDNP phenomenon can readily be interpreted in terms of the radical pair mechanism (RPM), which accounts for almost all phenomena observed during the analysis of thermally or photochemically induced reactions of spin‐correlated radical species in solution and relies on the nuclear spin‐dependent modulation of singlet–triplet mixing rate constants. To understand how the nuclear spin dependence of singlet–triplet mixing leads to CIDNP, the reaction scheme shown in Figure 4 can be considered. In a first step, a spin‐correlated radical pair in its triplet state is formed as a result of an electron abstraction reaction between the triplet‐excited electron acceptor P and the ground state electron donor Q. Held together by a cage of solvent molecules, this triplet radical pair then has two alternative ways to react: (i) the two partner radicals can either diffuse apart and get scavenged at a later stage to form so‐called escape products (here PX and QY) or, alternatively, (ii) react via a back electron transfer reaction to yield so‐called recombination products, i.e., the initial diamagnetic starting compounds. In the absence of any hyperfine interactions between the unpaired electron and magnetically active nuclei of the same compound, both pathways have an almost equal chance to occur. If, however, the radical electron's precession frequency is modulated by these hyperfine interactions, the probability of the radical pair to recombine will be spin‐dependent.

**FIGURE 5:**
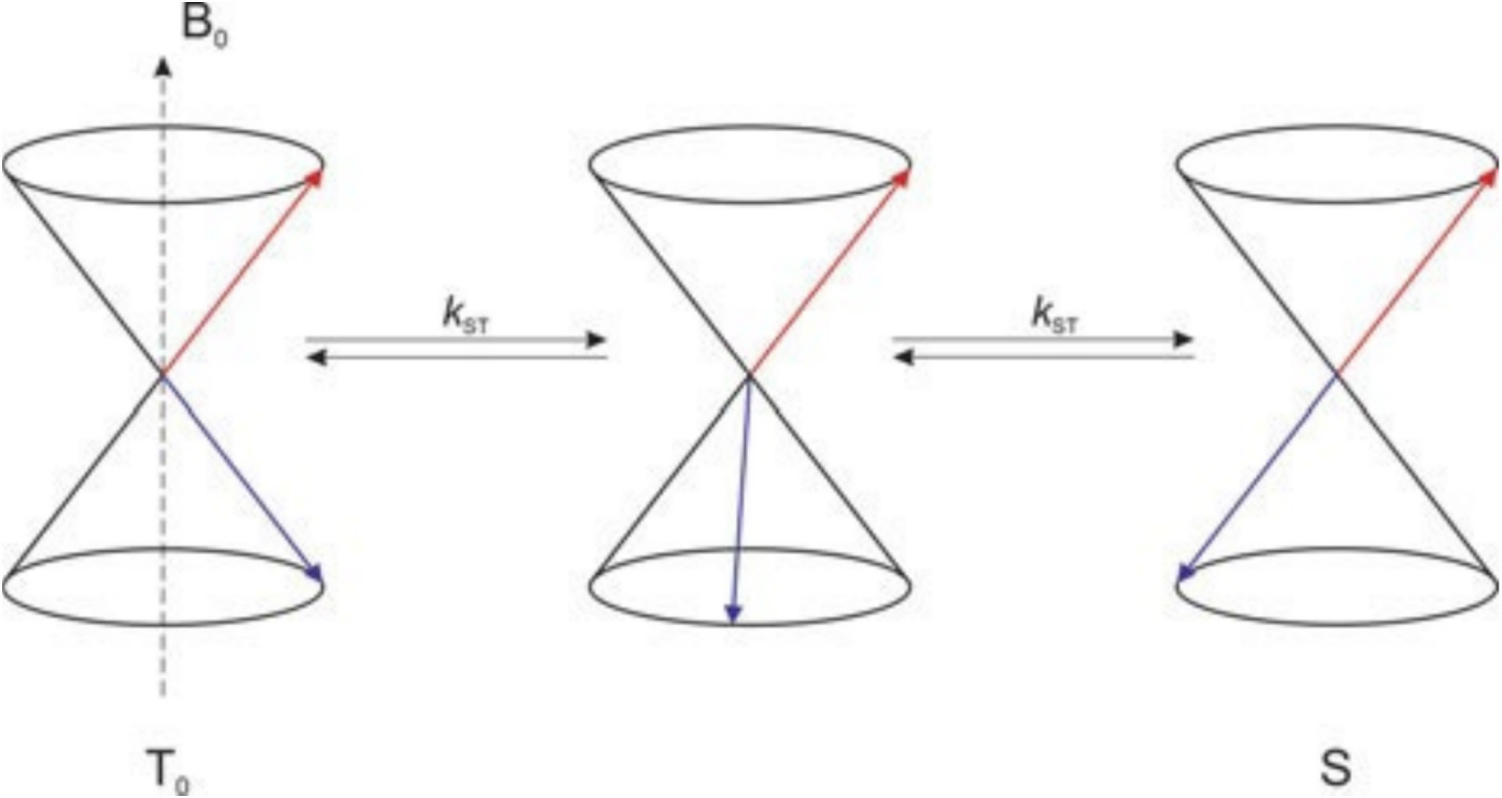
Vector model representation of singlet–triplet mixing. The cones represent the individual electron spin states and the arrows the electron spins. The intersystem crossing rate (*k*_ST_) is determined by the precession frequency difference of the two radical electrons.

The recombination likelihood of a radical pair depends strongly on the electronic spin state of the pair, with reaction usually only possible from a singlet state as the Pauli principle has to be obeyed. Hence, the triplet radical pair must first convert to a singlet state in order to proceed via the recombination route. If singlet–triplet mixing is faster when a nucleus on Q is in its α state, then a nuclear spin sorting process will take place, with triplet radical pairs containing a nucleus in its α state more likely to interconvert into a singlet state and then recombine. On the other hand, triplet pairs comprising nuclei of opposite spin quantum number will have a greater probability of diffusing apart and react via the escape route depicted in Figure 4. As a consequence of this nuclear spin selective chemistry, recombination products will contain an excess of *α*‐spin state nuclei and hence show an absorptive, i.e., positive, NMR enhancement upon application of a 90° read‐pulse and, conversely, escape products will possess an excess of *β*‐spin state nuclei leading to an emissive, i.e., negative, enhancement in the NMR spectrum. In exactly the same way, when singlet–triplet mixing is slower for radical pairs comprising α‐spin nuclei or for an initial radical pair formed from a singlet precursor, the situation is reversed, leading to positive enhancements in the magnetic nuclei of the escape products and negative enhancements in those of the recombination products (Figure 5).

## 3 Experimental Methods

### 3.1 Light Sources and Coupling Methods

#### 3.1.1 Light Sources

Most of the early work on photo‐CIDNP employed either lamps of various types or, on the other hand, rare gas, e.g., argon ion, lasers [13]. Two different types of light sources, however, are now predominantly used to perform continuous‐wave photo‐CIDNP experiments on small organic compounds as well as biomolecules in solution: (i) laser diodes or, alternatively, (ii) light‐emitting diodes (LEDs). Other suitable systems are rarely used these days, mainly because both laser diodes and LEDs are very easy to set up, portable, and available across a wide range of wavelengths and output powers, typically between a few milliwatts and several watts. Also, they take up very little laboratory space and are much more cost‐effective than, for example, the much bulkier and very expensive continuous‐wave rare gas lasers.

Laser diodes (LDs) are voltage‐driven semiconductor lasers in which a diode is directly pumped with electric current to create lasing conditions. The choice of semiconductor material determines the wavelength of the emitted beam, which in today's laser diodes ranges from infrared (IR) to ultraviolet (UV). LDs are often used in the form of laser diode modules or ‘diode lasers’, i.e., packages containing one or more laser diodes, often combined with appropriate optics and electronics. Such modules are much easier to use than bare laser diodes because they combine additional elements to facilitate their operation. For example, diode lasers may include additional optics outside the laser cavity, such as a beam collimator or beam shaper, as well as means for coupling the light into an optical fibre, essential for their use in photo‐CIDNP experiments (see below). In some cases, they are also encapsulated—a feature that greatly enhances laser safety—and may contain means for temperature stabilisation and a laser diode driver to provide the necessary power. Most commercially available LDs have an output power of 500 mW or more, classifying them as ‘Class 4’ lasers. ‘Class 4’ lasers can damage the eyes and burn skin and materials, particularly dark and/or lightweight materials, at close range. They should therefore be used with extreme care, and extensive laser safety measures and specific personnel training must be implemented.

Light‐emitting diodes (LEDs), on the other hand, are optoelectronic semiconductor devices that generate light via electroluminescence, where the wavelength of the emitted radiation depends on the energy band gap of the semiconductors used. Although the fundamental process of light generation is the same as for laser diodes, LEDs do not exhibit laser action, i.e., they do not usually exploit stimulated emission. Unlike a laser, the light emitted by an LED is neither spectrally coherent nor highly monochromatic and has low spatial coherence. Consequently, it cannot approach the very high intensity characteristics of a laser, and the beam quality is relatively low. The emission bandwidth is typically a few tens of nanometers, i.e., much wider than that of laser diodes, which means that the temporal coherence of the light emitted by an LED is also much lower than that of a laser. LEDs are recommended as a suitable light source for use in photo‐CIDNP NMR experiments due to their ease of use, low cost and availability in a wide range of wavelengths. To achieve satisfactory results, high‐power LEDs connected to a single‐stage electronic circuit switched directly by the pulse sequence used to acquire photo‐CIDNP data—i.e., via a TTL signal of the NMR spectrometer's time control unit—should be preferred [22].

#### 3.1.2 Light Coupling

Both laser diodes and LEDs can emit a light beam into free space. In order to conduct photo‐CIDNP NMR experiments ‘in‐situ’, however, it is essential to couple the optical output into a narrow‐diameter fibre to deliver the light into the NMR tube located in the interior of the NMR spectrometer. Whereas LEDs require optical fibres featuring a rather large numerical aperture (NA ≥ 0.5), laser diodes can be coupled to optical fibres having NAs as small as 0.22 and core diameters between 0.4 and 1.0 mm. Routinely, it is recommended to use 1‐mm core diameter multimode optical fibres (numerical aperture (NA): 0.5; wavelength range: 300–1200 nm), inserted into the NMR magnet from above via the magnet bore, for most purposes. A suitable FC/PC, FC/APC or, alternatively, SMA adapter needs to be available to connect the optical fibre directly to a diode laser. When using such a solution, it is advisable to purchase an appropriately terminated optical fibre which, in addition, is flatly cleaved at its far end. If the diode laser does not have a direct fibre connector, an additional launch system is required to couple the light into the fibre. Coupling of an LED light source to an optical fibre, on the other hand, is achieved via placing the LED in direct contact with the end of the optical fibre, flatly cleaved at both ends. This is a viable option because the light‐emitting area of the LED is approximately of the same size as the cross‐sectional area of the optical fibre core. Thereby, losses due to either the highly divergent LED output or the use of additional optics are minimised, allowing much higher light intensities to be transmitted. Higher coupling efficiencies can be achieved by cutting off the silicone lens of the LED, as this brings the optically active part of the LED into even closer contact with the end of the fibre [22].

### 3.2 NMR Insert and Fibre End Treatment

#### 3.2.1 NMR Insert

**FIGURE 6:**
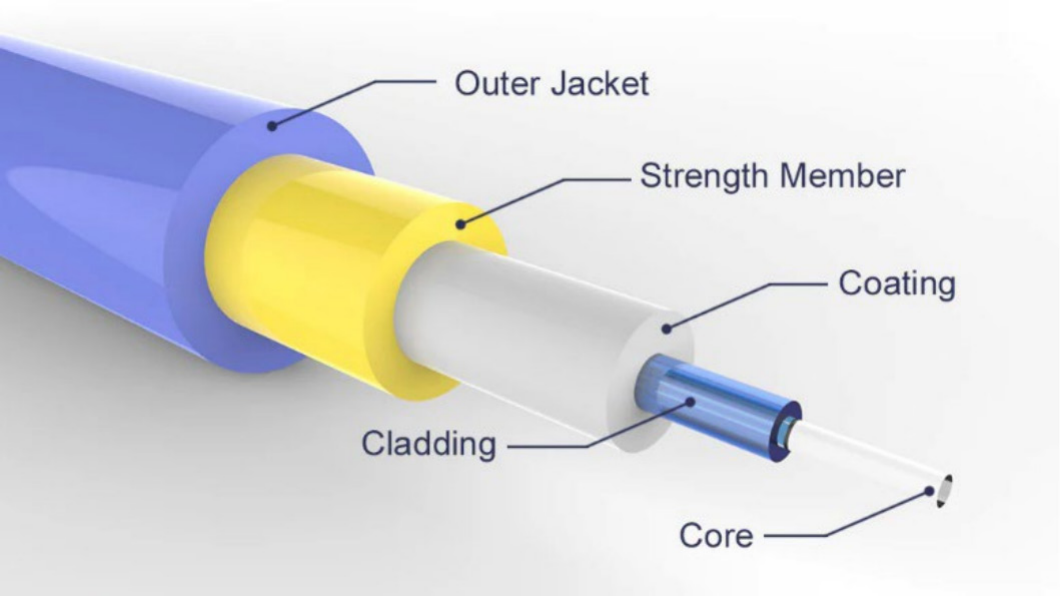
The different components of an optical fibre: While the core of an optical fibre provides the path for the electromagnetic radiation to propagate, its cladding prevents light from exiting the core and being absorbed by the rest of the cable. The coating, or buffer, protects the core and cladding and provides strength. When the fibre is manufactured into a cable, the next layer is comprised of a material, e.g., aramid, which gives strength to the cable and helps prevent damage from stress. The whole package is then encased in a jacket. This outer jacket provides a final layer of protection and also adds strength to the fibre.

**FIGURE 7:**
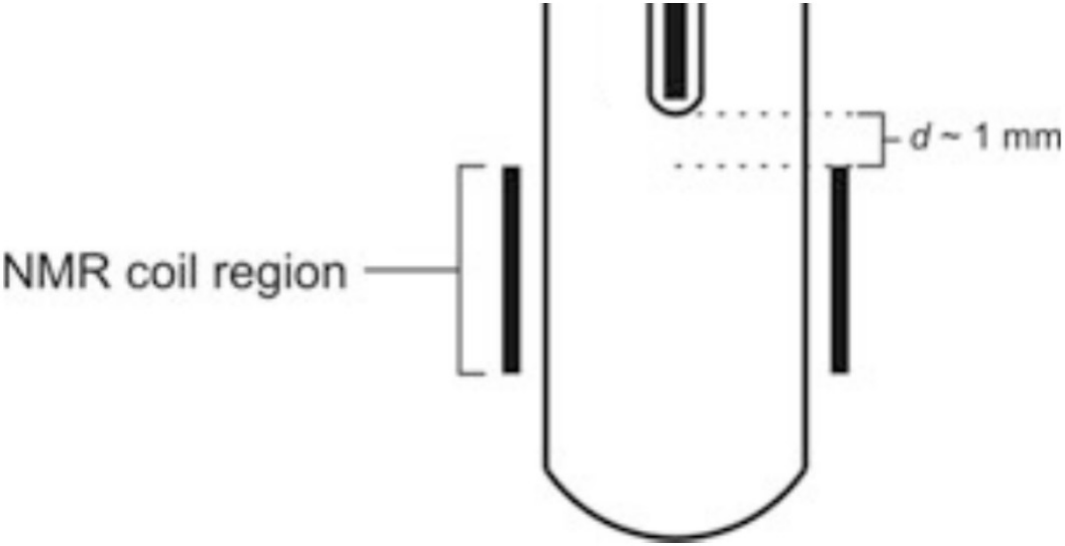
Graphic representation showing how to position the NMR insert containing the optical fibre prior to performing photo‐CIDNP experiments requiring ‘in situ’ illumination. The tip of the insert should be placed approximately 1 mm above the upper r.f. coil threshold using the depth gauge provided by the spectrometer's manufacturer (see text).

The output end of the optical fibre may either be dipped into the sample—although this can lead to problems with sample contamination, excessive local heating effects and damage to the tip of the fibre—or installed into the probe body from below [29]. Most commonly, however, the fibre is mounted in a coaxial glass insert—e.g., Wilmad WGS‐5BL (stem length: 50 mm; SP Wilmad LabGlass, Vineland, NJ, USA), typically used for internal chemical shift referencing—fitted into the sample tube (see below) [30]. The latter is an attractive option, although it precludes sample spinning. For the output end of the optical fibre to fit into the stem of the insert, i.e., up to the tip of the insert, its terminal portion—approximately 55 mm in length—must be stripped of all layers except the fibre core itself using appropriate fibre‐optic termination tools (Figure 6). When attached to a standard 7‐in. NMR tube^1^ containing the sample solution, the top part of the insert is held in place by a regular NMR tube cap with a small hole drilled in the centre. This arrangement can be further secured by wrapping several layers of parafilm around the tube‐insert interface before lowering the sample into the NMR spectrometer. Alternatively, Torres et al. have presented a 3D‐printed adaptor scheme using an O‐ring for a secure fibre‐NMR tube connection [25]. This design is cost‐efficient, safe, and relatively simple to obtain, and its use is therefore recommended. Prior to performing photo‐CIDNP experiments, the end of the insert should be positioned approximately 1 mm above the top of the coil region, using the depth gauge provided by the NMR spectrometer's manufacturer (Figure 7). This will minimise *B*_0_ field inhomogeneities while ensuring adequate illumination of the active sample volume.

The sample is then manually lowered into the magnet using the optical fibre as a means of controlling its descent into the coil region of the magnet. The arrangement of the sample tube and insert inside the NMR magnet, i.e., prior to performing a photo‐CIDNP experiment, is shown in Section 4.1. As mentioned above, the use of this setup precludes sample spinning due to the presence of the optical fibre. However, this is not a problem with modern NMR spectrometer software and hardware, as efficient automated shimming procedures are routinely available.

#### 3.2.2 Fibre End Treatment

**FIGURE 8:**
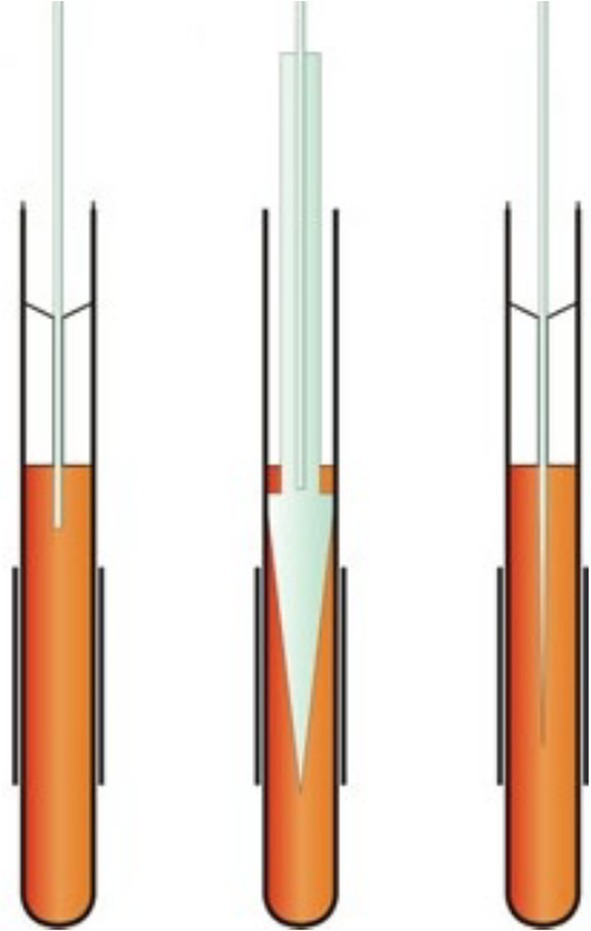
Schematic drawings of different fibre‐optic‐assisted NMR ‘in situ’ sample illumination methods, e.g., for photo‐CIDNP experiments, where a light guide is introduced from above into the bore of the magnet: (left) illumination from above using an optical fibre held in a coaxial glass insert; (middle) a variant in which the light is distributed by means of a pencil‐shaped tip insert; (right) another variant using a stepwise tapered optical fibre [31].

Light exiting the flat end of an optical fibre is emitted in a cone‐shaped manner. Ideally, however, the entire volume of the sample solution should be uniformly illuminated to maximise sensitivity and to avoid concentration and temperature gradients. These problems are most severe for optically dense samples and cannot be adequately addressed using a standard tip design. To overcome this problem, a simple and inexpensive method of illumination from above was developed, whereby laser light is distributed along the axis of the NMR tube via a tapered optical fibre (Figure 8) [31]. Whereas illumination from above the coil results in an exponential decrease in light intensity from the top of the sensitive r.f. receiver coil region to the bottom, a tapered tip—optical path length 20 mm in the former case vs. approx. 3 mm in the case of the latter—results in almost uniform illumination. In addition, the approach requires no modification of the probe, leads to less than a 5% loss of filling factor and causes minimal degradation of spectral resolution. Alternatively, it has been shown that roughening of the fibre tip, either by sandblasting or manually by treatment with sandpaper, can also result in uniform illumination of the active sample volume [22]. It should also be noted that fibreless illumination protocols have recently become available for conducting photo‐CIDNP experiments, allowing an almost uniform illumination of the relevant regions of the sample tube as well [24].

### 3.3 Photosensitisers

**FIGURE 9:**
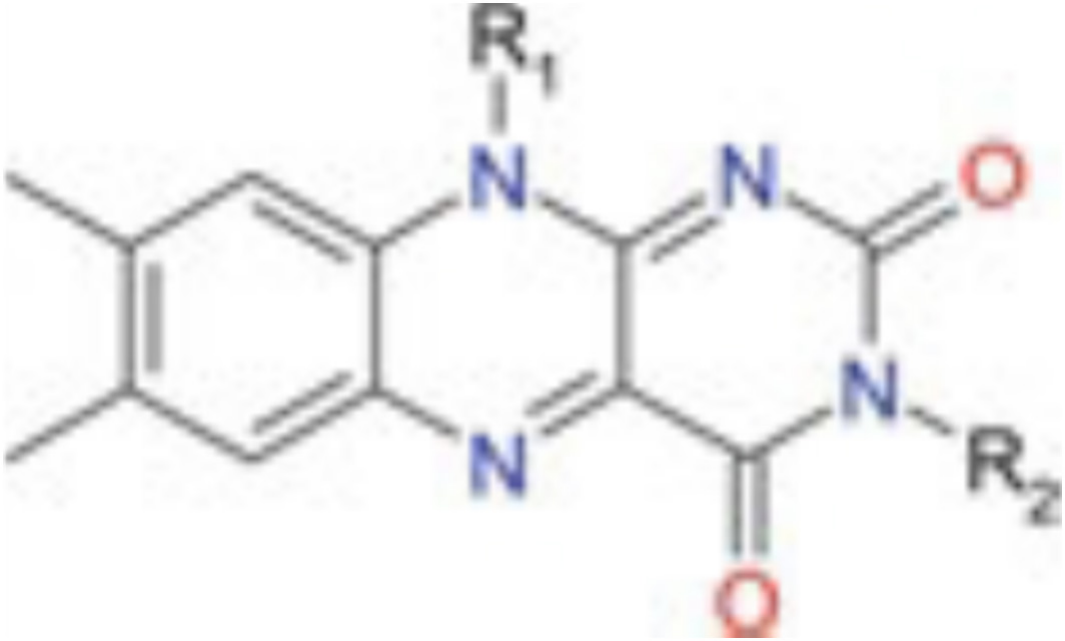
Structural representation of flavin derivatives frequently used as photosensitizers in photo‐CIDNP experiments of amino acids and proteins. The different chemical composition of flavin derivatives is characterised by the substituents ‘R_1_’ and ‘R_2_’.

The nuclear polarisation generated during a photo‐CIDNP experiment is due to a photochemical reaction between an electron or hydrogen donor molecule—i.e., the photo‐CIDNP substrate to be hyperpolarised—and a photoexcited electron or hydrogen acceptor dye molecule, i.e., the photosensitiser. The most commonly used photoactive dyes for performing photo‐CIDNP experiments are flavins, e.g., lumiflavin, riboflavin, flavin mononucleotide (FMN) and flavin adenine dinucleotide (FAD). Their general structure, based on the tricyclic isoalloxazine core, is shown in Figure 9. The chemical, biochemical and photochemical properties of flavin compounds are well characterised and understood. They are widely distributed in nature due to their ability to undergo facile, reversible one‐ and two‐electron redox reactions. The three common redox states are the (oxidised) flavin itself (F), the flavosemiquinone radical, the fully reduced flavohydroquinone (FH2) and their deprotonated forms. Flavins are commonly found as coenzymes, often noncovalently bound to proteins and are responsible for the electron transfer properties of flavoproteins, e.g., hydrogenases, oxidases and monooxygenases. Flavins also act as cofactors in cryptochromes, a class of blue light‐sensitive flavoproteins involved in animal and plant magnetoception as well as the stabilisation of their circadian rhythm [32, 33].

Of all the known flavin derivatives, FMN has emerged over the years as one of the most versatile and reliable photosensitisers for performing photo‐CIDNP experiments on small and large (biomolecular) substrates. The UV–VIS absorption spectrum of FMN is characterised by broad absorption bands occurring in the near UV and in the blue and green regions of the electromagnetic spectrum at wavelengths (λ) of approximately 375 and 450 nm, respectively. While the absorption at a wavelength of λ = 514 nm is rather weak, the extinction coefficient ε at λ = 488 nm is greater than 10^4^ dm^3^ mol^−1^ cm^−1^. Therefore, excitation into this band leads to efficient and rapid formation of the triplet state (quantum yield ca. 0.5) when using a light source emitting at this specific wavelength.^2^ Furthermore, FMN is water soluble and induces photo‐CIDNP in, for example, tryptophan (Trp), tyrosine (Tyr), histidine (His) and/or methionine (Met) side chains via electron or hydrogen abstraction to yield a spin‐correlated radical pair in a cyclic photochemical reaction, giving rise to the observed nuclear polarisation. Empirically, it has been found advantageous to use flavin‐based photosensitisers at a concentration of approximately 0.2 mM (±0.1 mM). This concentration of photosensitiser used in each photo‐CIDNP experiment represents a compromise between a sufficiently low optical density of the sample solution to allow its uniform illumination and, on the other hand, a high yield of triplet‐excited photosensitiser molecules present upon photoinduction.

In addition, several other dyes have been investigated as alternatives to flavins [13]. For example, xanthenes (fluorescein, rose bengal or eosin) polarise tyrosine but not tryptophan or histidine when irradiated with, e.g., green light [34, 35, 36, 37]. Of these, rose bengal does not yield any measurable enhancements in water—due to the presence of oxygen—but rather in methanol. Other xanthene‐based dyes exhibiting higher *g*‐factors, e.g., AT12, produce even stronger photo‐CIDNP enhancements [38]. Methylene blue and some porphins also generate CIDNP in tyrosine. In addition, *p*‐methoxyacetophenone, irradiated at a wavelength λ ~ 250 nm, gives strong CIDNP from Tyr and Trp but little enhancement of His. Quinoxaline, excited at the same wavelength, gives strong signals for tyrosine and histidine but not for tryptophan [39]. Broadhurst et al. tested a range of quinones, aromatic ketones, aza‐aromatics and a variety of dyes and sensitizers [40]. The aza‐aromatic compounds 2,20‐dipyridyl (DP, 2,20‐bipyridine) and, to a lesser extent, 2,20‐bipyrazine produce the largest enhancements when irradiated with UV light. DP is often used in microsecond time‐resolved CIDNP experiments using, for example, xenon‐chloride excimer lasers to investigate cancellation effects caused by recombination or degenerate exchange [41]. Studies of the reactions of the triplet state of DP with Trp, Tyr and His show that the rate constants, and hence the CIDNP, are strongly dependent on the protonation state of both DP and the amino acid side chain. Although Trp and Tyr residues are strongly enhanced, the polarisation of His is weaker and varies with pH. In addition, fluorescein, which absorbs strongly in the visible region of the electromagnetic spectrum at a wavelength of approximately 500 nm, has been identified as a very promising photo‐CIDNP dye that produces strong nuclear enhancements in tyrosine, tryptophan and many other aromatic compounds but not in histidine. In particular, fluorescein has been shown to facilitate photo‐CIDNP data collection at extremely low sample concentrations [42]. In summary, the most efficient water‐soluble photo‐CIDNP sensitisers reported in the literature are FMN, fluorescein and AT12. Also, they absorb in the visible region of the electromagnetic spectrum, which is safer and causes less wear and tear on the optical fibre over long periods of time as compared to using UV‐emitting light sources.

### 3.4 1D Photo‐CIDNP Pulse Sequences

#### 3.4.1 ^1^H Photo‐CIDNP—Difference Spectroscopy

**FIGURE 10:**
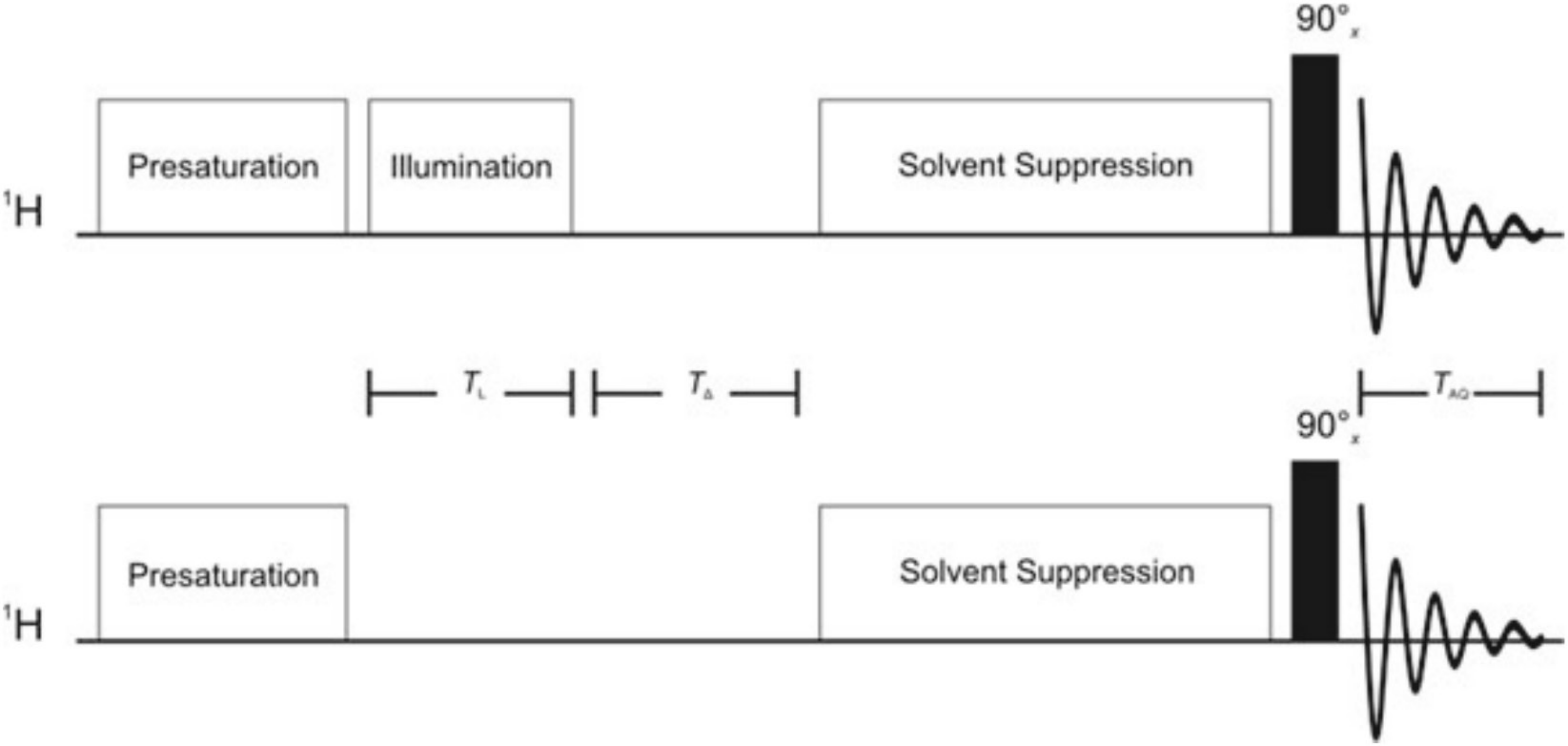
The basic pulse sequence for photo‐CIDNP difference spectroscopy. The ‘light’ (top) and ‘dark’ (bottom) halves of the sequence are identical except for the illumination period. The delay *T*_D_, not shown here, is defined as the time between the acquisition of the ‘light’ and ‘dark’ spectra. The implementation of sequence elements for presaturation and/or solvent suppression is optional (see below).

The basic ^1^H photo‐CIDNP experiment is relatively straightfoward to realise as it only requires a short illumination period (*T*_L_) to be incorporated into a standard pulse‐acquire NMR sequence at an appropriate position, i.e., before the read‐pulse is applied and the NMR spectrum is acquired. The simplest method of observing the laser light‐induced photo‐CIDNP effect is by difference spectroscopy [43]. As shown in Figure 10, a free induction decay (FID) without (‘dark’) and, subsequently, another one with (‘light’) a previous laser flash—typical irradiation times (*T*_L_) are between 0.1 and 1.0 s—are acquired and then subtracted from each other, resulting in a difference spectrum that exclusively shows resonances representing nonequilibrium spin state distributions induced by the CIDNP effect. Difference spectroscopy highlights the features that change between the ‘light’ and ‘dark’ spectra but does not provide any new information, and hence, the signal‐to‐noise ratio (SNR) found in the two spectra is degraded by a factor of √2 in the difference spectrum. However, this loss of SNR is, in most cases, negligible given the additional signal enhancement due to the CIDNP effect. Moreover, it can be reduced by acquiring more ‘dark’ than ‘light’ transients, followed by appropriate scaling of the ‘dark’ spectrum prior to subtraction [44, 45]. A short delay (*T*_Δ_ ~ 5 ms) between the light and the r.f. pulse is typically used to allow time for diffusing (free) radicals to recombine and thus avoid paramagnetic broadening of the signals [34]. In addition, sample heating happening during the acquisition of the ‘light’ spectrum can lead to line broadening and small chemical shift deviations (CSDs), resulting in subtraction artifacts in the difference spectrum. It is therefore advisable to allow a sufficiently long delay of about 10–20 s between the acquisition of the two spectra, i.e., ‘dark’ and ‘light’, for temperature equilibration. Alternatively, CIDNP‐specific presaturation techniques or dedicated photo‐CIDNP pulse sequences can be used (see below).

#### 3.4.2 Light Source Triggering

In most cases, an NMR pulse sequence designed to read out the nonequilibrium nuclear spin magnetisation generated by the photo‐CIDNP effect comprises almost identical sequence elements as compared to its ‘thermal’ counterpart. The most striking difference between the two is the implementation of an illumination period, i.e., a sequence element responsible for triggering the light source, which is physically connected to the console of the NMR spectrometer (see below). Normally, this element consists of a command to switch on the light source, a delay during which the NMR sample is illuminated, and, subsequently, another command to switch the light source off. For Bruker NMR spectrometers equipped with either an AVANCE III, AVANCE III HD or an AVANCE IV (NEO) console, this element can be conveniently introduced into the pulse sequence code by using one of the four unassigned, i.e., spare, TTL 'output' lines, which employ voltage‐gated real‐time control pulses (RCP) to trigger an external device, e.g., a light source or shutter. The appropriate pulse sequence syntax as well as abbreviations for addressing each of the four TTL lines individually can be found in the text file *Avance.incl*, which contains a series of macro definitions and is supplied with each ‘TopSpin’ installation (command: ‘edpul Avance.incl’):


/*;trigger outputs 1-4*/
#define TTL1_LOW setnmr3|28
#define TTL1_HIGH setnmr3^28
#define TTL2_LOW setnmr3|29
#define TTL2_HIGH setnmr3^29
#define TTL3_LOW setnmr3|30
#define TTL3_HIGH setnmr3^30
#define TTL4_LOW setnmr3|31
#define TTL4_HIGH setnmr3^31




Logically, the choice of the correct pulse sequence command used for addressing the respective TTL line on the NMR spectrometer's console will depend on which of the four pins the light source is physically connected to (see below). For example, for a light source connected to ‘pin I1’ of the T0‐connecter on the IPSO (Intelligent Pulse Sequence Organiser) of an AVANCE III HD NMR console, the pulse sequence code for a simple one‐dimensional proton NMR pulse‐acquire scheme comprising an illumination period ‘d33’—i.e., the photo‐CIDNP ‘light’ spectrum—will be as follows, taking into account macro definitions for ‘trigger on’ (TTL1_LOW) and ‘trigger off’ (TTL1_HIGH) as specified in the file *Avance.incl*:


1 ze
2 30m
 d1
 **TTL1_LOW**
 **d33**
 **TTL1_HIGH**
 p1 ph1
 go=2 ph31
 30m mc #0 to 2 F0(zd)
exit
ph1=0 2 2 0 1 3 3 1
ph31=0 2 2 0 1 3 3 1




#### 3.4.3 Presaturation Methods

**FIGURE 11:**
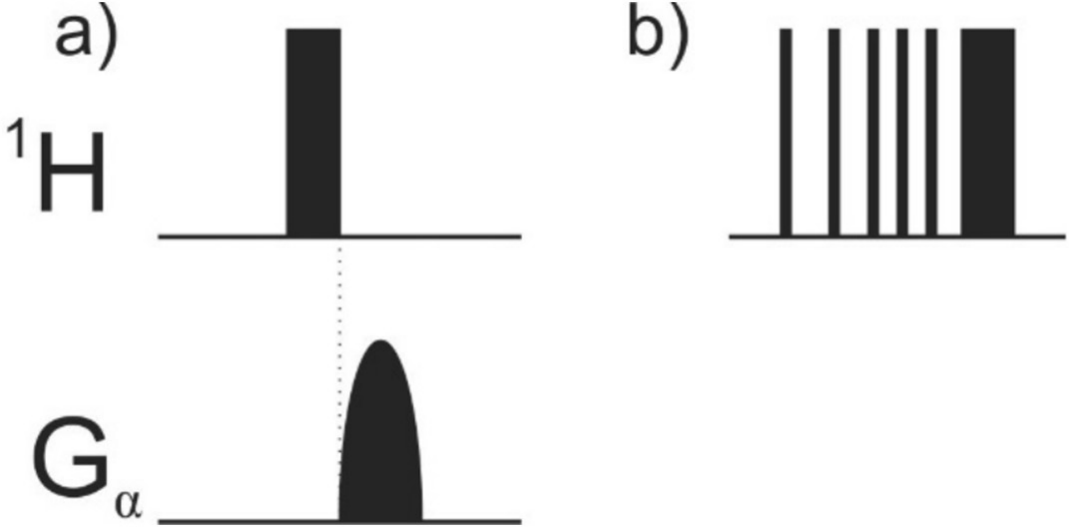
Presaturation techniques: (a) the crush pulse comprising a π/2‐pulse followed by a short ‘crush’ gradient (G) pulse to dephase the coherent magnetization and (b) the multiple pulse train. Provided that appropriate hardware is available, the gradient in the crush pulse is applied along the magic angle.

The generation of CIDNP results in a nuclear spin system exhibiting non‐Boltzmann spin state distributions. This so‐called nuclear polarisation is detected in the NMR spectrum either as signals of enhanced absorption, opposite phase or both. To observe these polarization signals more clearly and to provide an alternative method avoiding the problems of subtraction artifacts associated with the difference spectroscopy technique described above, it is advisable to presaturate the whole spectrum prior to the light flash [43]. The use of such a presaturation technique minimises the signals observed from any unpolarised nuclei. The two possible ways of presaturating the whole spin system prior to the laser flash comprise either a π/2‐pulse followed by a short ‘crush’ gradient (G) pulse to dephase the coherent magnetization or, alternatively, a train of r.f. π/2‐pulses of random phase with an interpulse delay determined by a converging geometric series (Figure 11). The former sequence is complete in approximately 10 ms as opposed to 1 s for the latter and is hence particularly attractive when a short delay between two subsequent spectra is required. Both methods effectively suppress background signals by several orders of magnitude; hence, for the technique to be successful, the length of the laser flash must be small compared to the respective spin–lattice relaxation times. Presaturation can replace difference spectroscopy when *T*_L_ and *T*_D_ are small compared to the shortest *T*_1_ of the sample. The only disadvantage of the presaturation method becomes obvious when working under conditions where nuclear spins are able to return very quickly to thermal equilibrium. Since proteins, for example, are large macromolecules, they tumble very slowly in solution; hence, their rotational correlation times are rather long. An implication of this is that nuclear spin relaxation becomes more efficient the slower a molecule tumbles; hence, given that the length of a laser pulse applied during a CIDNP experiment is long compared to the typical relaxation time of a small protein, significant relaxation can be expected to occur during the laser pulse. This means that the effect of the presaturation applied to the spins becomes less pronounced. Since this effect can be counteracted by using difference spectroscopy methods and also because subtraction artifacts in difference spectra can be reduced by presaturation, the two techniques are commonly combined under conditions of fast spin–lattice relaxation.

#### 3.4.4 ^1^H Photo‐CIDNP—Direct Acquisition

For the direct acquisition of ^1^H photo‐CIDNP hyperpolarisation NMR data, a specific one‐dimensional ^1^H photo‐CIDNP NMR pulse sequence developed by Hore and co‐workers can be used, which yields a ‘pure’ photo‐CIDNP spectrum, thus rendering the subsequent subtraction of photo‐CIDNP ‘light’ and ‘dark’ spectra superfluous [46]. The experiment combines presaturation of (thermal) background magnetization by a series of composite π/2‐pulses, each followed by a defocusing field gradient. Subsequent gated illumination during a grid of π‐pulses with a prescribed timing causes the background magnetisation to disappear at those moments in a pulse sequence when photo‐CIDNP‐derived magnetisation is to be sampled or transferred. By shifting the illumination intervals within such a grid, the sign of the polarisations can be inverted without affecting the evolution of background magnetisation, allowing a further strong suppression of residual background by a phase cycle. Accordingly, utilising this pulse sequence allows the acquisition of photo‐CIDNP NMR spectra that are virtually free from background magnetisation, thus avoiding the sensitivity loss and subtraction artifacts associated with difference spectroscopy. Although the use of this pulse scheme is recommended as a general tool for the acquisition of ^1^H photo‐CIDNP data, it has proven to be particularly beneficial for the acquisition of hyperpolarisation data of (complex) mixtures whose spectra often suffer from subtraction artifacts when photo‐CIDNP difference spectroscopy is employed [26].

#### 3.4.5 Heteronuclear Photo‐CIDNP

Standard one‐dimensional pulse sequences with or without proton decoupling can be used to obtain heteronuclear, e.g., ^13^C, photo‐CIDNP NMR data provided that they contain statements addressing the triggering of the light source (see above). Accordingly, 1D inverse‐gated or power‐gated proton‐decoupled as well as coupled pulse sequences can be used to observe the photo‐CIDNP effect on ^13^C. If the photo‐CIDNP substrates are not isotopically enriched in ^13^C, difference spectroscopy is normally not necessary, since, in most cases, the thermal NMR signal will be below the noise level of the NMR spectrum, and, to a first approximation, the photo‐CIDNP ‘light’ spectrum will be virtually identical to the photo‐CIDNP difference spectrum. When ^13^C‐enriched compounds are used, difference spectroscopy and/or presaturation techniques must be applied, preferably in combination with using a destructive phase cycle acting on the thermal, i.e., background, magnetisation. As an example, the Bruker pulse sequence code for a 1D photo‐CIDNP ^13^C{^1^H}‐pulse scheme based on the ‘zgig’ sequence featuring inverse‐gated proton decoupling is shown below. Similar prerequisites apply to the acquisition of photo‐CIDNP data of heteronuclei other than ^13^C.


1 ze
 d11 pl12:f2
2 30m do:f2
 d1
 **TTL1_LOW**
 **d33**
 **TTL1_HIGH**
 p1 ph1
 go=2 ph31 cpd2:f2
 30m do:f2 mc #0 to 2 F0(zd)
exit
ph1=0 2 2 0 1 3 3 1
ph31=0 2 2 0 1 3 3 1




### 3.5 NMR Spectrometer

#### 3.5.1 RCP Pin Connections

The power supply of the light source, or alternatively, the shutter device, must be physically connected to the NMR spectrometer console via a standard BNC cable in order to be correctly triggered by the appropriate pulse sequence statements mentioned above. When using either a Bruker Avance III or III HD console, the BNC cable needs to be connected to an unassigned pin of the console's IPSO T0‐connecter via using a ‘6P300 COAXIPack BNC’ adapter. If a Bruker Avance IV (NEO) console is used, a dedicated RCP adapter box featuring four BNC connectors, each representing one of the four unassigned RCP ‘output’ channels, needs to be used. The adapter box is connected to the console's ‘TRIG RCP I/O’ port via a standard RJ45 (Cat. 5e) patch cable. A list of all available unassigned TTL output pins can be found in the ‘IPSO 19’ and ‘IPSO AQS’ user manual located in the Bruker Advanced System Handbook (BASH Table 1a and Table 1b).

**TABLE 1a:** Excerpt of a list of pin assignments of TTL real‐time control pulses (RCP) available at the front side connector ‘T0’ of the IPSO 19″ unit of a Bruker Avance III or III HD console (source: Bruker ‘IPSO 19’ & ‘IPSO AQS’ User Manual). The four outputs shown are not used by any internal process of the console electronics and can therefore be utilised to trigger an external unit, e.g., a light source.

Source/Destination	FIFO Word/Bit Position		tctrl output reg.	setnmr0(#)	setnmr3(#)	setnmr4(#)	Layout Name	Direction	NMR		
	A	B							Meaning	T0	BSMS
MED	—	30	T1(12)	—	28	—	TCU27	out	Customer specified	**I1**	—
MED	—	31	T1(13)	—	29	—	TCU28	out	Customer specified	**I2**	—
MED	—	32	T1(14)	—	30	—	TCU29	out	Customer specified	**I3**	—
MED	—	33	T1(15)	—	31	—	TCU30	out	Customer specified	**I4**	—

**TABLE 1b:** BNC pin connections of ‘output’ TTL real‐time control pulses (RCP) available from a Bruker RCP adapter box connected to a Bruker AV4 (NEO) console. The four output channels are not used by any internal process of the console electronics and can therefore be utilised to trigger an external device, e.g., a light source.

Pin	Signal AV4 SCU
**2**	RCP_TTL_OUT(1)
**4**	RCP_TTL_OUT(2)
**6**	RCP_TTL_OUT(3)
**8**	RCP_TTL_OUT(4)

#### 3.5.2 Autosampler Operation

NMR spectrometers equipped with autosampling capabilities, e.g., Sample Case (Plus), Sample Xpress (Lite), SampleMail and SampleJet, can also be used for conducting fibre‐optic‐assisted photo‐CIDNP experiments. However, the direct access to the bore of the magnet via the ‘Bruker Sample Transport System’ (BST) must be provided in order to lower the sample into the spectrometer (see below). In most cases, this is achieved either by deactivating the device (Sample Case, Sample Case Plus) or, alternatively, by entering ‘manual mode’ (Sample Xpress (Lite), SampleMail and SampleJet). In ‘manual mode’, NMR samples can be inserted directly into the magnet bore, thereby bypassing the autosampler altogether. In this case, NMR samples must be placed in a standard spinner. In any case, it is recommended to consult the autosampler's manual for detailed, step‐by‐step instructions on how to either disable the equipment or, alternatively, enter ‘manual mode’. Recently, Wüster et al. presented a setup that couples an optical fibre with a helium‐ or nitrogen‐cooled cryogenic probe from below the NMR magnet via the probe's flow‐cell accessory port. This arrangement enables the high‐throughput automated measurement of photo‐CIDNP experiments as it is compatible with commercially available autosamplers. As such, it can also be used for other light‐coupled NMR experiments requiring high throughput [29].

### 3.6 Photodegradation

During photo‐CIDNP reactions, the flavin molecule is susceptible to photoreduction, leading to significant degradation or ‘bleaching’ of the dye, as the photoreaction underlying the photo‐CIDNP mechanism is not perfectly cyclic. This, in turn, can cause problems during continuous or multiple laser flash illumination, resulting in a gradual loss of polarisation as the concentration of oxidised flavin is depleted. For example, when studying the reaction of lumiflavin with the amino acid tryptophan, it was shown that the gradual loss of polarisation is due to both photodegradation of the flavin molecule and degradation of the amino acid itself [47]. Bleaching of the solution seems to be the more serious problem in this case, as CIDNP intensities are sensitively dependent on the optical density of the sample. In addition, the concentration of the flavin is, in most cases, about one order of magnitude lower than that of polarisable substrate molecules. The most likely cause of this photochemically induced reduction is the disproportionation of flavosemiquinone radicals, as indicated by microsecond time‐resolved CIDNP studies [48, 49]. However, flavin photochemistry is inherently complex, and hence, additional factors may contribute to the degradation of the dye molecule. In particular, a number of intramolecular and intermolecular light‐induced reduction, addition and dealkylation mechanisms have been proposed, with the predominant reaction pathway chosen depending on factors such as the type of solvent, pH, buffer composition and other parameters [50]. Furthermore, primarily formed products may themselves undergo a variety of secondary photolytic or thermal reactions, resulting in a highly complex sample mixture.

Several attempts have been made to combat the problem of photo‐CIDNP dye degradation: (i) Since bleaching occurs predominantly in the irradiated part of the NMR tube, (manual) sample mixing between acquisitions can be used to replenish the flavin [44, 45]. (ii) Manipulation of the molecular oxygen (O_2_) concentration may also be helpful: O_2_ efficiently reoxidises FH2 to F but is consumed in the process. Therefore, removal of O_2_ by degassing accelerates photobleaching. A high concentration of molecular oxygen, on the other hand, strongly attenuates the initial polarisation due to, amongst other things, quenching of photoexcited triplet flavin [47]. (iii) Another way of trying to overcome the problem of photodegradation is to spin the NMR tube rapidly between flashes so as to create a vortex in the solution [44, 45]. This reintroduces oxygen and brings fresh flavin into the irradiated region. However, fibre‐optic illumination is precluded using this approach. (iv) Other ways of increasing the lifetime of the flavin dye have also been explored, including the use of mechanical mixing devices and alternative chemical oxidation methods such as the use of hydrogen peroxide (H_2_O_2_) as an oxidising agent [51, 52]. For example, a PTFE transfer line passing through the coaxial insert can be used to withdraw and then inject a portion of the solution into the NMR tube between signal acquisitions. If the solution is reintroduced sufficiently quickly, the ensuing turbulence will cause efficient mixing and reintroduction of oxygen [51]. A more straightforward method is the addition of an oxidising agent, e.g., hydrogen peroxide (H_2_O_2_), which efficiently converts flavosemiquinone to its fully oxidised form. Although H_2_O_2_ is known to act as an oxidising agent of thioether, e.g., methionine, and thiol groups, e.g., cysteine, no apparent chemical modification of the protein hen egg‐white lysozyme (HEWL), containing two solvent‐inaccessible methionine residues, was detected using standard parameters, i.e., 298 K, pH 7 and 10 mM H_2_O_2_ [52]. (v) Lee and Cavagnero recommend the addition of a ‘tri‐enzyme mix’, which reduces the extent of photodegradation and, in particular, enhances photo‐CIDNP in experiments requiring long‐term data collection. This method allows efficient regeneration of FMN while minimising irreversible photodegradation. As molecular oxygen is known to be involved in many photodegradation pathways, O_2_ is first depleted in the NMR sample by the addition of glucose and the enzymes glucose oxidase (GO) and catalase (CAT). This enzyme system is widely used to minimise photodegradation in fluorescence microscopy. The resulting decrease in photo‐CIDNP—given that FMN cannot be efficiently regenerated from FH2 (see above)—is counteracted by the additional introduction of nitrate reductase (NR), an FH2‐oxidising enzyme. The resulting tri‐enzyme system significantly improves photo‐CIDNP performance while reducing the extent of irreversible sample photodegradation [53]. Also, the addition of low‐micromolar concentrations of either 2‐mercaptoethylamine or, alternatively, ascorbate can significantly reduce photodamage to the sample resulting in higher photo‐CIDNP enhancements [54].

## 4 Acquisition of Photo‐CIDNP NMR Data—A Step‐by‐Step Guide

The acquisition of a standard, i.e., one‐dimensional, ^1^H photo‐CIDNP NMR spectrum forms the starting point of every photo‐CIDNP study, both to check sample conditions and to test the general feasibility of hyperpolarising NMR‐active nuclei of, for example, a biomolecule of interest. Once set up, the measurement of a basic 1D CIDNP spectrum of a molecule or a mixture of molecules in aqueous solution is usually straightforward and fast. The sample concentration can be the same as or lower than that used for conventional NMR experiments, and the number of scans required for an acceptable SNR can often be as low as 8 or 16, depending (mainly) on the sample conditions. A small amount of FMN stock solution (c ~ 10 mM) is added directly to the NMR sample tube, resulting in a final dye concentration of approximately 0.2 mM. A preliminary ‘steady‐state’, i.e., equilibrium, photo‐CIDNP spectrum will provide general information on, for example, the solvent accessibility as well as the general polarisability of the target substrate(s) of interest. In the following, critical step‐by‐step information on the preparation, setup and performance of such solution photo‐CIDNP NMR experiments is provided.

### 4.1 Sample and Setup Preparation

The preparation of an aqueous photo‐CIDNP NMR sample—recommended to be carried out using a standard 1.5‐mL Eppendorf ‘safe‐lock’ tube—is relatively straightforward. First, the substance to be analysed, dissolved in D_2_O, is added to the tube. If the molecule of interest contains exchangeable ^1^H nuclei, e.g., amide protons, a solvent mixture comprising 10% D_2_O and 90% H_2_O must be used. Otherwise, potentially polarisable labile ^1^H nuclei may not be detected in the resulting photo‐CIDNP NMR spectrum due to exchange of these protons by deuterons. A small amount of an aqueous FMN stock solution (c ~ 10 mM) is then added to give a final FMN concentration of 0.2 mM in a total volume of 600 μL (see Scheme S1). Sample volumes of less than approximately 550 μL cannot be tolerated using standard NMR tubes as, in this case, the tip of the coaxial glass insert is not in direct contact with the sample solution. Smaller sample volumes down to approximately 350 μL can be illuminated using susceptibility‐matched ‘Shigemi’ NMR tubes. The sample is then mixed and subsequently centrifuged using a vortex mixer and a table‐top centrifuge, respectively. Finally, the sample solution can be transferred from the Eppendorf tube into a thin‐walled 7‐in. NMR tube (e.g., Wilmad 507‐PP‐7) using a long‐necked (length: 230 mm) Pasteur pipette. Care must be taken not to contaminate the inner wall of the NMR tube with liquid during the sample transfer, as this would render the subsequent insertion of the coaxial glass insert significantly more difficult.

**FIGURE 12:**
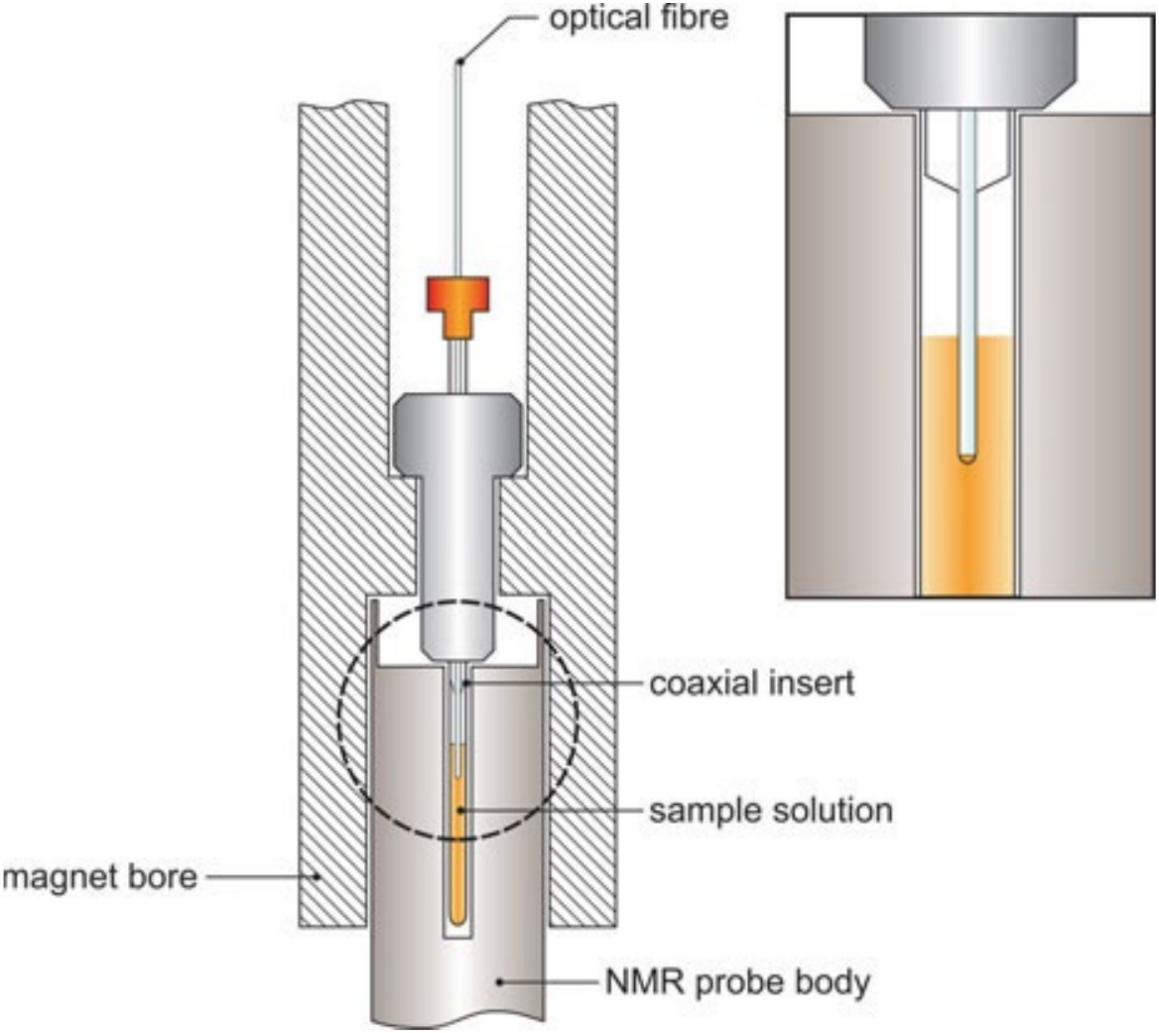
Schematic representation of the experimental setup inside the NMR spectrometer prior to performing solution state photo‐CIDNP NMR experiments. In particular, the correctly positioned NMR tube containing the sample solution (orange), the coaxial glass insert and the optical fibre are highlighted.

Once the sample solution has been transferred to the NMR tube, the coaxial glass insert holding the optical fibre in place can be carefully inserted into the NMR tube. The correct positioning of the insert in the tube is achieved using the depth gauge provided by the NMR spectrometer's manufacturer (see Section 3.2.1). Last, several layers of parafilm can be wrapped around the tube‐insert interface—n.b., the top part of the insert is held in place by a regular NMR tube cap with a small hole drilled in the centre—to fix the position of the insert with respect to the NMR tube's centre line. The whole assembly can then be transferred to the NMR spectrometer and lowered into the magnet by hand, using the optical fibre as an aid. Obviously, the presence of the optical fibre, which is attached directly to the NMR tube by means of the coaxial glass insert, prevents the use of the spectrometer's sample lift. The lock signal of the NMR spectrometer should be observed during this process to ensure that the sample tube has reached its correct position within the NMR spectrometer (Figure 12). It is therefore recommended that the NMR spectrometer be locked to a (standard) NMR sample containing the same deuterated solvent before inserting the photo‐CIDNP sample of interest. The NMR spectrometer can then be subjected to standard locking, tuning/matching and shimming procedures.

### 4.2 Photo‐CIDNP NMR Data Acquisition

Prior to the acquisition of photo‐CIDNP NMR data, a thermal one‐dimensional ^1^H NMR spectrum should be set up and acquired. This spectrum will be used as a reference for all subsequently collected photo‐CIDNP data. In addition, many of the sequence parameters optimised for the acquisition of the reference spectrum, e.g., digital resolution of the time domain (TD), spectral width (SW), transmitter offset frequency (O1) and 90° pulse width (P1), can also be used at a later stage for the collection of photo‐CIDNP data. Sequence parameters that need to be adjusted for the acquisition of photo‐CIDNP spectra are the receiver gain (RG) and the interscan delay (D1). For the acquisition of ^1^H photo‐CIDNP data, D1 should be conservatively set to a slightly longer value, e.g., 5 s, to allow for temperature equilibration between scans. For slower relaxing nuclei, e.g., ^13^C, this will need to be modified accordingly. As photo‐CIDNP leads to the observation of enhanced absorptive NMR signals—apart from also producing emissive NMR lines—the receiver gain (RG) used to acquire photo‐CIDNP spectra should be set slightly lower than the RG value used to acquire the thermal spectrum. This, however, is only relevant if the NMR signals representing the analyte molecules to be hyperpolarised are very intense in the thermal spectrum. If the concentration is low, RG values close to or equal to those used to acquire the thermal NMR spectrum should ensure that no overflow of the ADC's receiver occurs during acquisition of the photo‐CIDNP spectrum. In addition, a suitable illumination period—typically between 0.1 and 1.0 s in duration—must be determined. However, the choice of the correct illumination time depends on the experimental requirements and the composition of the photo‐CIDNP sample to be studied. It is determined, amongst other things, by factors such as the optical density of the sample solution and the total number of ‘light’ scans to be acquired, and should therefore be chosen individually by the operator. In principle, shorter exposure of the sample to light will result in the generation of smaller amounts of triplet‐excited photosensitiser molecules per scan—and therefore smaller photo‐CIDNP enhancements—but will also reduce the risk of degradation of the photo‐CIDNP dye. Conversely, prolonged exposure of the sample to laser irradiation significantly increases the risk of photobleaching.

Once all acquisition parameters have been set up and tested, the photo‐CIDNP NMR spectrum can be acquired. If ‘difference spectroscopy’ is to be performed, data collection should begin with the acquisition of the photo‐CIDNP ‘dark’ spectrum with the light source turned off. Typically, the photo‐CIDNP ‘dark’ spectrum should be acquired, accumulating eight to 16 scans for ^1^H data acquisition.^3^ More scans should only be acquired if the concentration of the analyte(s) of interest is low or very low. For the acquisition of heteronuclear photo‐CIDNP data, the number of scans must be increased unless isotopically enriched compounds are being analysed. Subsequently, the photo‐CIDNP ‘light’ spectrum can be recorded using the same pulse sequence and identical acquisition parameters as before. In this case, of course, the light source must be turned on.

### 4.3 Photo‐CIDNP NMR Data Processing and Analysis

The processing and analysis of photo‐CIDNP NMR data is, in principle, identical to that of thermally acquired NMR data. First, the FIDs are Fourier‐transformed, followed by zero‐ and first‐order phase correction. When ‘difference spectroscopy’ is applied, both photo‐CIDNP ‘light’ and ‘dark’ spectra must be processed independently and then subtracted from each other. Prior to subtraction, NMR signals affected by hyperpolarisation have to be aligned. However, alignment of ‘light’ and ‘dark’ spectra is often difficult because temperature gradients in the sample caused by illumination lead to non‐linear CSDs in the photo‐CIDNP ‘light’ spectrum. Accordingly, it is sometimes difficult to perfectly align all the hyperpolarised NMR signals at the same time prior to subtraction and, as a result, subtraction artifacts often occur in the photo‐CIDNP difference spectrum. These often hamper the analysis of photo‐CIDNP data significantly. Direct acquisition of the photo‐CIDNP ‘net’ spectrum using specifically designed NMR pulse sequences is therefore recommended (see Section 3.4.4). Properly processed photo‐CIDNP spectra can be subjected to a variety of different methods of analysis. For example, signal enhancement factors (*ε*) of hyperpolarised nuclei can be determined by comparing the integral of the corresponding photo‐CIDNP signal with the thermal data obtained using identical acquisition parameters. Hyperpolarisation patterns of photo‐CIDNP signals can also be useful reporters of the nature of the radical species involved in the formation of the spin‐correlated radical pair responsible for the generation of photo‐CIDNP [55, 56].

## 5 Conclusions

Photo‐CIDNP NMR spectroscopy of both small molecules and macromolecular entities is a sensitive, extremely versatile and highly biocompatible NMR hyperpolarisation technique that can be used, amongst other things, to elucidate the surface structure of native and non‐native states of proteins, study protein‐ligand binding events or, alternatively, simplify NMR spectra of complex (bio)mixtures. Over the last 60 years or so, the technique has undergone significant methodological developments, allowing it to mature into a highly reliable spectroscopic tool, complementing other well‐established NMR hyperpolarisation methods. In this tutorial, aimed primarily at the nonexpert user, we have attempted to provide practical, hands‐on information on how to plan, set up and perform one‐dimensional ^1^H and heteronuclear photo‐CIDNP NMR experiments. In particular, strategies for selecting the appropriate experimental setup have been described, including aspects such as light source requirements and photosensitiser selection. In addition, examples of suitable one‐dimensional ^1^H and heteronuclear photo‐CIDNP pulse schemes were presented, photo‐CIDNP‐specific acquisition parameters explained, and practical advice on photo‐CIDNP sample preparation was given. Special attention as to how to acquire and analyse one‐dimensional photo‐CIDNP data in a meaningful way was also provided. We firmly believe that the future of the method is bright, and there is no doubt that further advances in the field, both methodological and experimental in nature, can be expected in the not‐too‐distant future. These will undoubtedly help to extend the applicability and success of the technique even further.

## Supporting information


**Scheme S1:** Schematic representation of the workflow applied to aqueous samples being subjected to NMR analysis via photo‐CIDNP using an NMR ‘in situ’ illumination setup as described in the text and a state‐of‐the‐art NMR spectrometer.

## Data Availability

Data sharing not applicable to this article as no datasets were generated or analysed during the current study.

## Endnotes

^1^
If a coaxial insert is utilised, it is essential to use thin‐walled NMR tubes, e.g., Wilmad 507‐PP‐7, as the insert can only be fitted into these.

^2^
To perform photo‐CIDNP experiments, it is essential to select the wavelength at which the light source emits radiation according to the absorption characteristics of the photosensitiser used in the photo‐CIDNP experiment.

^3^
As mentioned before, the loss of SNR associated with applying ‘difference spectroscopy’ can be mitigated by acquiring more ‘dark’ than ‘light’ transients, followed by appropriate scaling of the ‘dark’ spectrum prior to subtraction.
