# Supplementary material for: Continuous‐Wave (CW) Photo‐CIDNP NMR Spectroscopy: A Tutorial
Source: Magn Reson Chem. 2025 Sep 4;63(12):1020–34. doi: 10.1002/mrc.70031 (PMC12583242; doi:10.1002/mrc.70031)
Supplement: Supplementary file 1 — Scheme S1: Schematic representation of the workflow applied to aqueous samples being subjected to NMR analysis via photo‐CIDNP using an NMR ‘in situ’ illumination setup as described in the text and a state‐of‐the‐art NMR spectrometer. [file MRC-63-1020-s001.docx]

**Scheme S1:** Schematic representation of the workflow applied to aqueous samples being subjected to NMR analysis via photo-CIDNP using an NMR ‘in-situ’ illumination setup as described in the text and a state-of-the-art NMR spectrometer.

1. Photo-CIDNP sample preparation using a standard Eppendorf ‘safe-lock’ tube: (i) addition of 588 µL of sample solution using D_2_O (see text); (ii) addition of 12 µL of the FMN photosensitizer stock solution (c ~ 10 mM) to the Eppendorf tube to yield a final FMN concentration of ca. 0.2 mM and a final sample volume of 600 µL.

**ǀ**

1. Thorough mixing of the sample solution using a vortex mixer followed by table-top centrifugation.

**ǀ**

1. Transfer of the sample solution into a thin-walled 7-inch NMR standard tube (diameter: 5 mm) using a long-necked (230 mm) Pasteur pipette.

**ǀ**

1. Connection of the NMR tube with the coaxial glass insert carrying the optical fibre.

**ǀ**

1. Fixation of the tube-insert interface using parafilm (see text).

**ǀ**

1. Transfer of the arrangement comprising NMR tube and glass insert into the NMR spectrometer.

**ǀ**

1. Application of standard locking, matching/tuning, and shimming procedures.

**ǀ**

1. Acquisition of a ‘thermal’ one-dimensional ^1^H and or ^13^C NMR reference spectrum.

**ǀ**

1. If ‘difference spectroscopy” is applied (see text): acquisition of the photo-CIDNP NMR ‘dark’ spectrum.

**ǀ**

1. If ‘difference spectroscopy” is applied (see text): acquisition of the photo-CIDNP NMR ‘light’ spectrum.

**ǀ**

1. If ‘direct acquisition” is applied (see text): acquisition of the photo-CIDNP NMR “net” spectrum.

**ǀ**

1. Standard NMR processing & analysis as well as application of “difference spectroscopy”, if applicable.
